# Psychotherapy and Genetic Neuroscience: An Emerging Dialog

**DOI:** 10.3389/fgene.2018.00257

**Published:** 2018-07-17

**Authors:** Juan P. Jiménez, Alberto Botto, Luisa Herrera, Caroline Leighton, José L. Rossi, Yamil Quevedo, Jaime R. Silva, Felipe Martínez, Rodrigo Assar, Luis A. Salazar, Manuel Ortiz, Ulises Ríos, Paulina Barros, Karina Jaramillo, Patrick Luyten

**Affiliations:** ^1^Department of Psychiatry and Mental Health – East, Faculty of Medicine, Universidad de Chile, Santiago, Chile; ^2^Human Genetics Program, Institute of Biomedical Sciences, Faculty of Medicine, Universidad de Chile, Santiago, Chile; ^3^Department of Psychology, Faculty of Social Sciences, Universidad de Chile, Santiago, Chile; ^4^Center for Attachment and Emotional Regulation (CARE), Faculty of Psychology, Universidad del Desarrollo, Santiago, Chile; ^5^Center for Intercultural and Indigenous Research, Anthropology Program, Institute of Sociology, Pontificia Universidad Católica de Chile, Santiago, Chile; ^6^ICBM Human Genetics Program, Centre for Medical Informatics and Telemedicine, Faculty of Medicine, University of Chile, Santiago, Chile; ^7^Center of Molecular Biology and Pharmacogenetics, Department of Basic Sciences, Faculty of Medicine, Universidad de La Frontera, Temuco, Chile; ^8^Department of Psychology, Faculty of Education, Social Sciences and Humanities, Universidad de La Frontera, Temuco, Chile; ^9^Department of Psychiatry, Universidad de Valparaíso, Valparaíso, Chile; ^10^Ph.D. Program in Public Health, School of Public Health, Faculty of Medicine, Universidad de Chile, Santiago, Chile; ^11^Faculty of Psychology and Educational Sciences, KU Leuven, Leuven, Belgium; ^12^Research Department of Clinical, Educational and Health Psychology, University College London, London, United Kingdom

**Keywords:** gene–environment interactions, epigenetic modifications, subjective experience, psychopathology, psychotherapeutic change

## Abstract

Recent research in psychiatric genetics has led to a move away from simple diathesis-stress models to more complex models of psychopathology incorporating a focus on gene–environment interactions and epigenetics. Our increased understanding of the way biology encodes the impact of life events on organisms has also generated more sophisticated theoretical models concerning the molecular processes at the interface between “nature” and “nurture.” There is also increasing consensus that psychotherapy entails a specific type of learning in the context of an emotional relationship (i.e., the therapeutic relationship) that may also lead to epigenetic modifications across different therapeutic treatment modalities. This paper provides a systematic review of this emerging body of research. It is concluded that, although the evidence is still limited at this stage, extant research does indeed suggest that psychotherapy may be associated with epigenetic changes. Furthermore, it is argued that epigenetic studies may play a key role in the identification of biomarkers implicated in vulnerability for psychopathology, and thus may improve diagnosis and open up future research opportunities regarding the mechanism of action of psychotropic drugs as well as psychotherapy. We review evidence suggesting there may be important individual differences in susceptibility to environmental input, including psychotherapy. In addition, given that there is increasing evidence for the transgenerational transmission of epigenetic modifications in animals and humans exposed to trauma and adversity, epigenetic changes produced by psychotherapy may also potentially be passed on to the next generation, which opens up new perspective for prevention science. We conclude this paper stressing the limitations of current research and by proposing a set of recommendations for future research in this area.

## Introduction: A New Intellectual Framework for Understanding Mental Disorders and Change Mechanisms in Psychotherapy

From the second half of the 20th century onward, psychiatry has been strongly influenced by the idea that genetics determines human behavior. However, over the last decades, neurobiological research has revealed that the opposite is also true: human behavior may modify gene expression. The gene expression of our genome is not as stable and invariable as traditionally thought. Functional genomic studies have shown that the genome does not always produce the proteins that affect human behavior in the same manner; rather, it has been observed that many genes can be finely regulated in response to certain socio-environmental conditions. Moreover, several studies have shown that subjective experiences, such as perceptions of social isolation and rejection, may mediate the influence of the environment on the innermost and deeper biological processes, that is, on the expression of our genes (Slavich and Cole, 2013). Hence, new evidence supports the idea that gene–environment interaction shapes each individual brain (Kandel, 1998; Cappas et al., 2005; Kendler, 2005). These findings make it possible to expand evidence–based explanations of change mechanisms in response to psychotherapy beyond psychology to the realm of biology.

Generally, while the intersection of neurobiology and psychotherapy research is a fertile and expanding area (Gerber, 2012), the dialog between neurogenetics and psychotherapy is still in its infancy. In fact, the last edition of the standard handbook of psychotherapy research (Lambert, 2013a) includes no reference to genetics.

The aim of this review is to explore how genetic neuroscience may contribute to better understand the mechanisms of change in psychotherapy. We begin this paper by focusing on the findings of over six decades of psychotherapy research, advancing the idea that research must go beyond psychology and into biology if we want to overcome the so-called paradox of equivalence between the existing psychotherapeutic models and understand the specific mechanisms that sustain the psychopathology of each disorder, which would boost the development of treatments based on such mechanisms. We then review the complex relationship between environment and gene expression, which suggests that psychotherapy may modify brain and behavior through the modification of gene expression. A integrative multilevel approach including a focus on gene–environment interaction, epigenetic regulation, and subjective experience, is discussed in relation to susceptibility to mental disorders and purported mechansims of change in psychotherapy. We conclude this paper by proposing recommendations for future research and clinical applications.

## Psychotherapeutic Change: the “Paradox of Equivalence” and the Emerging Research Paradigm in Psychiatry

Basically, psychotherapy is an interpersonal process whose goal is to modify feelings, behaviors, attitudes, and cognitions that have been problematic for a person (the patient) seeking help from a trained professional (the therapist) (Strupp and Binder, 1984). This definition considers three fundamental aspects: first, that psychotherapy is a particular type of relationship whose course is determined by a series of interpersonal transactions; second, that this type of relationship occurs between a person seeking help for some type of emotional distress (the patient); and, finally, that the provider of this help (the therapist) is a professional who has received specific training (through supervisions, seminars, and personal therapy).

Along with the relief of psychic suffering, one of the most important goals of psychotherapy is the restoration of the patient’s social functioning, i.e., the ability to maintain stable and productive interpersonal relationships that promote physical and emotional health within the social environment in which the individual develops. In that regard, the most important change probably occurs not within the therapy but in the person’s ability to use and modify their own social environment (Fonagy et al., 2015).

Although more than 400 types of psychotherapy have been described, most of them are subtypes of major orientations: psychodynamic, behavioral, cognitive-behavioral, interpersonal, systemic, or strategic (Roth and Fonagy, 2005). After more than five decades of psychotherapy research, we now know that psychotherapy is effective, that, in general, there are no significant differences in effectiveness between different types of psychotherapy, that the specific technique used explains only 8% of the variance of the results, and that the most important generic factor of change is the so-called “therapeutic alliance” (Wampold and Imel, 2015). It is generally accepted that the effect size for psychotherapeutic treatment, as compared to no treatment, is around 0.80 (Lambert, 2013a; Wampold and Imel, 2015). An effect size of 0.8 means that nearly 75% of patients receiving psychotherapy feel better at the end of the therapy compared to those who did not receive it and improved on their own. Psychotherapy is more effective than many common medical interventions, has fewer side effects, and is more cost-effective. In the most prevalent mental disorders, psychotherapy is comparable in effectiveness to medication and has fewer side effects. In addition, psychotherapy has a prophylactic effect that medication lacks (i.e., relapse rates are lower when treatment is discontinued) (Wampold and Imel, 2015). Nevertheless, there is still much room for improvement: overall, about half of the patients do not achieve remission, about one third drop out early, and there has been no increase in effect sizes over the six decades during which these effects have been studied (Weisz et al., 2017).

But, along with this promising general data, during the last decades we witnessed the “battle of the paradigms” (Kendler, 2005) between different psychotherapeutic approaches, where each tried to show superiority over the rest. However, process and outcome research in psychotherapy has shown just marginal differences between psychotherapies of a different persuasion (Wampold and Imel, 2015). The search for treatment specificity in psychotherapy has had the same results as in psychiatry. In psychiatry, pharmacological treatments are not very specific. For example, antidepressant medications, such as selective serotonin reuptake inhibitors, are used to treat not only depression, but also a wide variety of anxiety disorders; in addition, they are prescribed to decrease emotional deregulation in severe personality disorders. Antipsychotic medications are also used not only in psychosis, but also in bipolar disorder and to decrease paranoid ideation in borderline personality disorders (BPD). The same goes for psychotherapeutic treatments. It is almost a rule that therapies that are developed as specific treatments, for example, for depression or BPD, are subsequently shown to be effective in a broad spectrum of disorders (Cuthbert and Insel, 2013).

One reason behind this is that research has targeted mental disorders defined according to DSM and/or ICD criteria. In recent decades, criticism of these diagnostic systems, particularly the DSM system, has increased. The central criticism points to the fact that DSM is a diagnostic system based upon the clinical presentation of signs and symptoms, with reasonable reliability but dubious validity. For example, the validity of the DSM-IV diagnosis of major depression, a highly prevalent disorder, has been criticized in many ways (Maj, 2012). For some, the Depression diagnostic threshold set by DSM-IV is too high, thus excluding many depressive states that do not differ from the major depression currently defined in other variables; for others, it is too low, so that milder cases diagnosed do not respond better to antidepressants than to placebo. The threshold of the number of symptoms for diagnosis has also been criticized. Subjects with a history of minor depression (i.e., 2–4 depressive symptoms with no previous history of depression, bipolar disorder, or dysthymia) are not distinguishable from MDD with 5 or more symptoms with respect to prognosis or other variables (Feighner et al., 1972; Kendler and Gardner, 1998). Furthermore, the ability of the operationalized diagnosis to distinguish depression from bereavement and life adjustment situations has been criticized. Based on DSM diagnostic criteria, more than 200 combinations of possible symptoms can be used to define a depressive episode. Thus, for some researchers, the studies performed to date do not provide conclusive evidence for the existence of depressive symptom dimensions or symptomatic subtypes (van Loo et al., 2012). In contrast, the psychopathological analysis of depressive symptoms emphasizes the need to consider specific clinical profiles that may be a consequence of diverse etiopathogenesis and, therefore, require differentiated treatments (Ghaemi et al., 2012). So, research in psychiatry faces the major challenge posed by this enormous clinical pleomorphism (Mann, 2010). Heterogeneous syndromes grouped into one disorder are highly likely to include several pathophysiological mechanisms. Considering this situation, the National Institute of Mental Health has launched the Research Domain Criteria Initiative (RDoC), whose aim is to “develop, for research purposes, new ways of classifying mental disorders based on dimensions of observable behavior and neurobiological measures” (Cuthbert and Insel, 2013, p. 4). The RDoC project proposes that future psychiatric and psychotherapeutic research should focus on systems underlying basic psychological capacities (such as reward neurocircuitry and the neural systems involved in self-representation, theory of mind, attachment/separation fear, and positive and negative valence systems), rather than on discrete DSM disorders. The RDoC proposes that the research process focus on psychobiological functions first and then move on to symptoms; not the other way around, as has traditionally been done. From this perspective, disorders are regarded as extreme cases of dysfunction of these systems, which orients nosology in a direction that is more dimensional than categorical, unlike the DSM approach (Cuthbert and Insel, 2013). Therefore, RDoC is a transdiagnostic approach. At first glance, RDoC seems to be a reductionist initiative insofar as it seems to regard mental diseases as brain diseases. However, a careful look shows a more sophisticated view. Actually, the RDoC recognizes the current developments in mental health that suggest that the causes of mental disorders can operate at different levels, such as genetic, neural, psychological, family, and social contexts. These etiopathogenic levels interact with each other in complex ways and affect the onset, course, and prognosis of mental disorders (Bolton, 2013; Cuthbert and Kozak, 2013). Thus, the RDoC is a dimensional, transdiagnostic, and multilevel approach that recognizes “bottom–up” causation as well as “top–down” causation.

However, the RDoC is not free from criticism (Weinberger et al., 2015). Two main criticisms have been leveled against RDoC: (1) Limited clinical applicability (Maj, 2014). Patients seek help due to symptoms and do not take functional domains into account. The object of psychiatry is the altered, *personal* experience associated with one’s own suffering or that of others. The study of this altered experience is the field of psychopathology as a discipline. Neuroscientific findings are only of interest inasmuch as they can help explain and treat this suffering. In this regard, the RDoC denies the psychopathological foundations of psychiatry (Parnas, 2014). Clinicians must decide whether or not the patient is ill; to this end, they evaluate symptoms and decide whether a certain level of symptomatic severity is the cut-off point between health and mental illness. A purely dimensional approach does not allow for this decision. Thus, the clinician will continue to need a classification system based on phenomenology. (2) The lack of evidence to support many of the RDoC constructs. At present, there is a huge explanatory gap in genetic research between (a) the statistical associations of genomic variants and (b) mental symptoms, traits, or specific disorders. Genetic pleiotropy, the multiple genes involved, and the tiny effect size of existing associations make it difficult to demonstrate causality (Jablensky and Waters, 2014).

In any case, the RDoC initiative represents an opportunity for research in the study of vulnerability factors involved in mental disorders and hence in a mechanism-based psychotherapy (Hershenberg and Goldfried, 2015; Luyten and Fonagy, 2017). The point of view of the RDoC initiative can shed new light on the ‘paradox of equivalence’ in psychotherapy research (Stiles et al., 1986): the so called ‘Dodo bird verdict’ (Luborsky et al., 1975), according to which ‘all psychotherapies have won, all are better than no treatment, but none has shown superiority over other.’ Wampold and Imel (2015) holds that the medical model in psychotherapy, defined as the assumption that the efficacy of psychotherapy is due to specific methods for the treatment of specific problems, has failed to explain the evidence that research has accumulated over more than half a century. However, at least two objections can be aimed at this statement: (1) The medical model venerates randomized controlled trials (RCTs) as the gold standard to make conclusions about research evidence, but RCTs use DSM diagnostics to select patients; thus, evidence yielded by RCTs may be flawed, as suggested by the foundations of the RDoC initiative (specific dysfunctions that specific interventions target are not revealed by DSM diagnostic insofar as DSM categories likely include groups of patients with different pathophysiological mechanisms); and (2) even if we accept that Wampold’s contextual model is correct—i.e., that the relationship between the therapist and the client that occurs in the *context* of a treatment is critical to the success of therapy—there is a lack of understanding of how the contextual ingredients of the therapeutic relationship really work; these ingredients being “the real relationship, the creation of expectations through explanation and agreement about the tasks and goals of psychotherapy, and the facilitation of psychologically beneficial processes of some kind” (Wampold and Imel, 2015, p. 256). Although decades of research on therapeutic processes have identified many characteristics of the client, the therapist, their interaction, and treatment activities that predict therapeutic results, such as the therapeutic relationship, catharsis, the warmth of the therapist, learning, changing expectations, mastery, and *common factors* between different therapies, among others, “there is little empirical research to provide an evidence-based explanation of precisely why treatment works and how the changes come about” (Kazdin, 2009, p. 419). If we accept mind/brain unity and the principles of the new research paradigm proposed by the RDoC, psychotherapy research has a shared agenda with the neurosciences.

Neuroimaging studies in psychotherapy that examine the patterns of brain activity associated with treatment response and those that examine changes in brain activity occurring during treatment currently allow to delineate neural models of psychotherapy action (Fournier and Price, 2014). However, at present, no evidence exists of the neurogenetic mechanisms underlying these neural models. While we have some ideas of what happens in the brain during psychotherapy, little is known about the molecular biology of these processes or the “dialog between genes and synapses” (Kandel, 2001). The mechanism whereby psychotherapy achieves its effect is highly likely to be quite different for genetically distinguishable groups of individuals. Research on the neurogenetics of psychotherapy is aligned with the RDoC initiative and makes it possible to go beyond the simple reordering of symptomatic constellations by establishing how known facts across genomic, environmental, endophenomic, and phenomic domains can be reassembled to identify groups of etiopathologically meaningful and empirically verifiable entities, remaining agnostic to traditional, phenotypic boundaries (Cuthbert, 2014).

Therefore, the aim of this paper is to review what we know about how these domains relate to psychotherapy. However, to understand the interdisciplinary challenge posed by the dialog between genetics and psychotherapy, we must first introduce some central concepts and findings of modern genetic neuroscience.

## Gene–Environment Relationship: Interaction and Correlation

Psychiatric disorders (PDs) are complex multi-gene disorders, likely with hundreds of susceptibility genes interacting with environmental factors such as stressful experiences (Gelernter, 2015). Thus, there is a growing need to identify genes and networks and to understand mechanisms and external factors related to normal and pathological behavior.

The complexity of the human genome is enormous. The human genome (3.2 gigabases, Gb) hosts about 25.000 protein coding genes. These genes are located in 23 pairs of chromosomes in the nucleus of the cell and in a short molecule of DNA (1.6 kilobases, kb) located in the mitochondria. The human genome exhibits various kinds of sequence variants in populations. They are estimated at approximately 84,000,000 single nucleotide polymorphisms (SNP), 3,000,000 short insertions/deletions (indels), and 60,000 structural variants (1000 Genomes Project CONVERGE consortium, 2015). Therefore, the genetic diversity in populations makes it possible to predict an immense variety of potential gene–gene interactions (GxG) in individuals.

The new DNA sequencing technologies (next generation sequencing, NGS) have made it possible to sequence thousands of exomes (coding DNA sequences of an individual), genomes (whole DNA sequence of an individual), and transcriptomes (collection of all the RNA molecules present in a cell type or population of cells). The resulting information has revealed that the genotype-phenotype relationship is far more complex than expected. Thus, one gene can be associated with multiple phenotypes (multifinality) while one specific phenotype can be caused by mutations in multiple genes (equifinality) (Cicchetti and Rogosch, 1996). Consequently, one specific mutation can have different effects on different individuals, which could be explained by different profiles of genetic variations in different individuals and under the influence of a variety of environmental factors.

Two major ways in which genes relate to the environment have been described (Caspi and Moffitt, 2006; Kendler, 2011): (1) gene–environment interaction (GxE) and (2) gene–environment correlation (rGE).

Gene–environment interaction occur when the effect of exposure to a given environment is conditioned by the genotype of the person and *vice versa*. In interaction models, the fundamental premise underlying the hypothesis of gene–environment interaction is the moderating role of genes in the effect of the environment on phenotype and, in the same way, the moderating role of exposure to different environments in the effect of genes on a phenotype. GxE explain why people respond differently to environmental factors (e.g., why certain individuals are more prone to depression after exposure to negative life events or why certain individuals with genetic risk are less susceptible to depression if they have been exposed to positive environments).

On the other hand, rGE refers to the reciprocal influence that can occur between genes and the environment; that is to say, genes can exert an influence on the characteristics of the environment and *vice versa*, which does not imply that an interaction exists. More specifically, it refers to the genetic differences determined by exposure to particular environments. According to this model –widely used in evolutionary biology– animals modify their environment through genetic programming with the aim of favoring adaptive phenomena. However, recursively, such environmental modification can also increase the risk of psychopathology. This happens when a personality trait with a strong genetic component (like impulsivity) favors involvement in adverse environments that can cause mental health problems. Three types of rGE have been outlined in the literature: (a) passive, (b) reactive, provocative, or evocative, and (c) active or selective (Kendler and Eaves, 1986). Passive rGE refers to situations in which children inherit not only a genetic constitution from their parents, but also the environment in which they have been raised (Plomin et al., 1997) (e.g., they inherit an athletic constitution and family sports habits). The association between genetically related individuals is a prerequisite for passive rGE. The terms evocative, provocative, or reactive rGE refer to the tendency of certain genetically influenced temperamental behaviors to elicit specific types of responses from people within their environment (e.g., a child with a difficult temperament is more likely to provoke negative parenting behaviors). Active or selective rGE is defined as the active generation of certain environments based on genetically determined behavior tendencies. It refers to the association between the genetic characteristics of the individual and the environmental niches that the individual selects or generates (for example, an intellectually curious child will tend to find intellectually rich environments, while a child with a behavioral disorder will look for peers with similar behaviors) (Plomin et al., 1997).

Correlation and interaction models are not mutually exclusive. A genetic polymorphism can be correlated with some traits that generate changes in the environment and interact with the environment to determine a phenotype. An example of such a mediational model is the finding that the short polymorphic allele in the promoting region linked to the serotonin transporter gene (5HTTLPR) correlates with neuroticism (Greenberg et al., 2000; Sen et al., 2004), which in turn has been shown to be associated with a tendency to interpret life events negatively (John and Gross, 2004) and therefore with higher rates of depression. A moderation model, for example, is exemplified by an early GxE study that reported that the risk for developing depression is augmented by the interaction between the 5-HTTLPR genotype with the number of stressful life events experienced (Caspi et al., 2003). This polymorphism, located in the promoter of the 5-HTTgene, is related to its transcriptional activity (Heils et al., 1996).

It is important to note that in recent years research in this field has moved from low-throughput genetic association studies, in which one or a few genetic loci are genotyped at a time (candidate genes), to high-throughput genome-wide association studies (GWAS) that include thousands of gene variants (CONVERGE consortium, 2015; Hou et al., 2016; Yu et al., 2016; Power et al., 2017). A recent GWAS meta-analysis of major depressive disorder (MDD) (Wray et al., 2018) revealed that: (1) the majority of associated loci are common genetic variants located in non-coding regions highly conserved in mammals; (2) several variants overlap between different PDs, for example MDD and Schizophrenia; (3) variants identified are associated with mild depressive symptoms in the general population; (4) many of these variants map to genes related to the prefrontal and anterior cingulate cortex, which are important areas in depression; and (5) variants map to genes expressed in neurons but not oligodendrocytes or astrocytes.

## Gene Expression Changes: Transcriptional Activity

The structural plasticity of the neural architecture of the brain is molecularly explained by gene expression changes related to normal development/differentiation and to response to environmental alterations. Accordingly, changes in brain function caused by PDs have been related to alterations in the expression of several genes in several brain areas (Aston et al., 2005; Kang et al., 2007, 2012; Tochigi et al., 2008; Chandley et al., 2014; Barde et al., 2016) including the hippocampus (Klok et al., 2011; Medina et al., 2013). Therefore, several efforts to characterize normal and abnormal gene expression profiles have been performed in cell-specific manner at multiple levels, including the epigenetic/epigenomic one, messenger RNA expression profiles (transcriptomes), protein expression profiles (proteomes), and metabolite pools produced during metabolism (metabolomes), among others. Most studies have focused on the transcriptome and epigenetic/epigenomic levels (Bakulski et al., 2016).

The methods performed to analyze transcript expression have evolved from single gene expression to whole transcriptome analyses (microarrays and RNA sequencing). For instance, high exposure to environmental stress can lead to mental illnesses such as bipolar disorder (BPD), MDD, and post-traumatic stress disorder (PTSD) (McEwen, 2004). Patients with these mental disorders exhibit altered transcriptional profiles in some brain areas (Ramaker et al., 2017). Stress affects gene expression through the action of glucocorticoids (GCs), lipophilic molecules released by the adrenal gland after stress exposure. GCs in target tissues such as hippocampus and hypothalamus cross membranes and bind and activate GC receptors (GR) in the cytoplasm. GR is a type of transcription factor that once activated translocates to the nucleus and binds DNA sequences known as glucocorticoid response elements (GRE), thereby activating the transcription of the target genes. The intensity and duration of stressors determine whether the response is adaptive or maladaptive (McEwen, 2007). Thus, exposure to an acute stressor activates several effects including enhanced memory of danger, adaptive immunity, and metabolic changes that adapt the organism for dealing with the threat (Rubin et al., 2014). On the other hand, more intense and/or longer instances of stress has negative effects, including memory impairment, cardiovascular disease, and metabolic syndrome (McEwen, 2007).

The study of transcriptomics has yet to produce conclusive results regarding the etiology of psychiatric traits, disorders, and/or response to drugs and psychotherapeutic treatments. This is partly explained by the complexity of studies of this type given the diversity of populations of neural cells, their microenvironments, the innumerable external environments to which they can be exposed, the time and intensity of exposure to a stressor, genetic backgrounds, and the various behavioral phenotypes that can be analyzed (Rubin et al., 2014).

In relation to individual genes, some findings are promising. For instance, one gene that has been studied in relation to response to stress and psychiatric traits is the brain derived neurotrophic factor (BDNF). After stress exposure, certain genes are regulated at the transcriptional level: the BDNF (Fumagalli et al., 2004) and tropomyosin receptor kinase B (TrkB), among others (Begni et al., 2017). Prenatal stress in rats or social defeat stress in mice reduced BDNF levels in the hippocampus and the prefrontal cortex (PFC) (Tsankova et al., 2006; Luoni et al., 2014), and reduced hippocampal TrkB levels (Dwivedi, 2009). Accordingly, cytokines that induce depressive-like behavior in animals also cause a significant reduction in BDNF expression (Guan and Fang, 2006; Song et al., 2013). Decreased serum and plasma levels of BDNF have been observed in depressed people and also in the hippocampus in postmortem studies (Dwivedi et al., 2003; Karege et al., 2005; Dunham et al., 2009; Lee and Kim, 2010), while the Val66Met polymorphism has been associated with PDs (Sen et al., 2003). Thus, it has been suggested that the BDNF could be involved in the adaptability to environmental conditions.

Since psychiatric traits are complex, this implies that many genes are involved; therefore, research should focus on networks rather than on individual genes. The study of transcriptomics is possible by using microarray and NGS technologies (RNAseq), since they make it possible to analyze many thousands of mRNAs simultaneously.

Transcriptomic studies in animal models have shown that both acute and chronic stressors induce changes in anxiety-like behaviors, hippocampal function, and changes in gene expression, although these effects are different depending on the type of stressor. For instance, the hippocampal transcriptional profile in response to acute stress differs depending on whether the animal was previously exposed to chronic stress, even if there was a recovery period (Sen et al., 2003; Verhagen et al., 2010, p. 3574). Thus, each stressful situation that occurs can alter the baseline, which also depends on the stage of development in which each experience occurs.

As previously mentioned, the diversity of neural cell populations and their continuous variation in gene expression make it difficult to understand the complexity of gene expression in the brain. Many efforts have been carried out to dissect different specific cells, including laser capture microdissection of subpopulations of cells from fixed tissues, fluorescence activated cell sorting (FACS) to isolate pure cell populations, expression of EGFP tag to isolate single cell subtypes using translating ribosomal affinity purification (TRAP), and a transcriptome *in vivo* analysis (TIVA) tag which makes it possible to evaluate *in vivo* the transcriptome profile of single cells (reviewed by Rubin et al., 2014). Thus, a growing amount of genome-wide gene expression data has been generated using differential technologies; nevertheless, results have not been well replicated. The lack of reproducibility could be explained by biological and technical factors, such as small sample sizes, clinical heterogeneity, comorbidities, differences in microarray or RNAseq platforms, and disparities between the statistical analyses used, among others. Therefore, biological findings need to be replicated in several studies before being accepted. In a recent article, the authors collected and compared information from 25 publications in which genome-wide expression data in depressed people was evaluated (Ciobanu et al., 2016). They focused on 16 different brain areas and 15 peripheral cell types. The rationale was to identify reproducible alterations in different reports. In the brain, they found 582 genes differentially expressed between depressed and control subjects, although only 57 were replicated. The diseases and functions with which these genes have previously been associated are neurological disease, connective tissue disorders, developmental disorder, psychological disorder, cell-to-cell signaling and interaction, cardiovascular system development and function, cellular assembly and organization, nervous system development and function, cellular development, carbohydrate metabolism, molecular transport, and small molecule biochemistry. In peripheral tissues, 21 differentially expressed genes were replicated.

Finally, it must be considered that transcriptional activity is not a direct indicator of protein synthesis. Protein synthesis also depends upon post-transcriptional and translational regulation mechanisms (Decker and Parker, 2012). For instance, the level of ribonucleoprotein complexes known as P-bodies (which are involved in gene expression mechanisms such as mRNA degradation, translation repression, and sequestration) is regulated by the BDNF in neurons (Schratt et al., 2004).

## The Nature-Nurture Relationship Revisited: From Genetic Vulnerability to Differential Sensitivity

The diathesis-stress model has generally been regarded as the etiopathogenic paradigm of most mental disorders. According to this model (Monroe and Simons, 1991; Patten, 2013), psychopathology originates due to the interaction of premorbid constitutional vulnerability (diathesis) and environmental aggressions (stress). Nevertheless, in the past few years it has been suggested that, rather than diathesis (understood as an organic predisposition), individuals have a differential susceptibility to environmental influences (Belsky and Pluess, 2009); this means that some individuals would not only be more vulnerable to the negative effects of an adverse environment but also extremely sensitive to the beneficial effects of a positive and nourishing environment, or even to the absence of adversity. According to the evolutionist models of “biological sensitivity to context” (Boyce and Ellis, 2005; Ellis et al., 2005) and “differentiated susceptibility” (Belsky et al., 2007), the differential effect of any given polymorphism can be seen as supporting the notion of plasticity rather than that of vulnerability to environmental stress (Brune, 2012). This model proposes that the same allelic variation that causes a predisposition to a PD when linked to an adverse environment could lead to a better than average response in the same domain when faced with favorable environmental conditions. Therefore, although individuals who are more “sensitive” to environmental stimuli are likely to be the most gravely affected by stressors, they are also likely to be better prepared for responding to positive stimuli (Belsky et al., 2007). Furthermore, considering that genetic polymorphism differentially renders individuals “susceptible to plasticity” regarding environmental stimuli (Brune, 2012), it can be argued that, from an evolutionary perspective, allelic variation grants a selective advantage if external contingencies have been beneficial (Wurzman and Giordano, 2012).

Recent decades have witnessed a clear shift in the study of psychopathology from models emphasizing either genetic (Hong and Tsai, 2003) or environmental (Brown and Harris, 1978) factors to models incorporating various relationships between the genome and the environment (Rutter, 2007; Uher, 2008; Dick, 2011), including cultural variables such as individualism/collectivism and gene–culture coevolution (Chiao and Blizinsky, 2010; Way and Lieberman, 2010). For instance, with regard to depression, much research has focused on interactions between environmental factors and polymorphism of the serotonin transporter gene promoter region (5-HTT), following the aforementioned study of Caspi et al. (2003) demonstrating that individuals with one or two copies of the short allele of the 5-HTT promoter polymorphism exhibited more depressive symptoms, diagnosable depression, and suicidality in relation to stressful life events than individuals homozygous for the long allele. Although these findings led to a renewed focus on the role of the environment and stress (Hammen, 2005) and early and later adversity in particular when seeking explanations to vulnerability for depression, especially among genetically predisposed individuals (Heim and Nemeroff, 2001; Heim et al., 2008; Risch et al., 2009), considerable methodological limitations remain (Leighton et al., 2017). In addition, it has been difficult to replicate an interaction between 5-HTTT and stress, and a recent meta-analysis reported only very modest effect sizes (Bleys et al., 2018).

The importance of explaining the pathogenesis of psychopathology based on the differential susceptibility model is that the prevention, diagnosis, and treatment of disorders changes. This model allows us to hypothesize that patients carrying prosocial alleles will respond better to a treatment involving social interaction and learning, such as psychotherapy. Consistently, short allele carriers respond less well to pharmacotherapy than patients carrying the long allele (Serretti et al., 2007). Indeed, if a potentially disadvantageous gene variant is maintained at a high prevalence, this might imply that natural selection has not been able to eliminate the variant because its effects on the phenotype are expressed only under certain environmental conditions and/or perhaps even because it confers an advantage under particular environmental conditions. The importance of including recent and positive events in GxE studies is that transforming the environment into a positive one, whether at a personal level (i.e., by encouraging prosocial behaviors and psychotherapy interventions) or at a sociocultural level (i.e., by lobbying for a wider, more positive environment for populations), could have positive outcomes, especially for more sensitive individuals.

In this vein, some studies have focused on the question of whether being a carrier of ‘plasticity alleles’ has an impact on psychotherapeutic response with some conflicting results. Brody et al. (2009) evaluated the effects of a family intervention designed to reduce risk behaviors among vulnerable children. Results showed that those classified as with “genetic risk” for being short allele carriers were the ones who benefited most from the program. These participants engaged in risky activities such as alcohol consumption, drug use, and early sexual activity substantially less often than long allele homozygotes and control subjects. Bryant et al. (2010) studied patients diagnosed with PTSD and demonstrated, contrary to expectations, that individuals carrying the short allele displayed a worse response to cognitive behavioral therapy (CBT) than patients homozygous for the long allele. Another study (Kohen et al., 2011) evaluated the response to psychosocial interventions in post-stroke depressed patients and found that, among patients carrying the short allele of 5HTTPLR, psychosocial treatment had a large effect that was not evident for patients homozygous for the long allele. Eley et al. (2012), reported preliminary results showing that children with an anxiety disorder carrying the short-short (SS) genotype were significantly more likely to respond to CBT than those carrying a long allele (SL/LL). A control group not receiving CBT was lacking and the association only emerged at follow-up. Bockting et al. (2013), could not corroborate the preliminary finding that 5HTTLPR is associated with response to CBT. They randomized 187 referred recurrently depressed adult patients in the Netherlands to either a brief CBT module designed to prevent recurrence or treatment as usual. The primary outcome was time to recurrence, assessed prospectively over 5.5 years. In this study, 5HTTLPR was not significantly associated with treatment response. Recently, the reported association between 5HTTLPR genotype and outcome following CBT could not be replicated in child anxiety disorder (Lester et al., 2016). The authors reported that children homozygous for the short allele showed more positive treatment outcomes, but with small, non-significant effects. In short, different allelic configurations can explain different patient responses to the same therapeutic interventions.

To summarize, the evidence regarding the complex relationship between genes and the environment has led to the development of etiopathogenic models beyond the classical paradigm of vulnerability to stress. Moreover, at the interface between the environment and genes, various processes occur which can be understood as a biological way of encoding the impact of life events on an organism (Isles and Wilkinson, 2008), serving as a molecular bridge between “nature” and “nurture” (Tammen et al., 2013). These processes are of an epigenetic nature.

## Epigenetic Regulation and Mental Health

The term epigenetics was first used by Conrad Waddington in the 1950s to refer to the mechanisms whereby the genotype resulted in a particular phenotype during embryonic development (Jamniczky et al., 2010). Although since then multiple attempts have been made to reach an agreed definition (Bird, 2007), at present it is considered that an epigenetic trait is a stable and heritable phenotype resulting from chromosomal changes without any alterations in the sequence of nucleotides (Berger et al., 2009). Thus, epigenetics refers to all mechanisms that regulate the genome through modifications that do not involve an alteration in the DNA sequence, including DNA methylation and hydroxymethylation (Suzuki and Bird, 2008), a number of histone modifications (Kouzarides, 2007), and the regulation of gene expression by non-coding RNAs [ncRNAs, e.g., long non coding RNAs (lncRNAs), and microRNA microRNAs (miRNAs)] (Wei et al., 2017). These mechanisms make it possible to condense the genome in a minute three-dimensional (3D) space (the nucleus), but retaining the capacity of interaction with the machinery that regulates gene expression. Epigenetic changes have three key features: (1) they depend on the environment (Zhang and Meaney, 2010), (2) are heritable, that is, can be transmitted to offspring (Daxinger and Whitelaw, 2012), at least to the first three generations, and (3) are dynamic throughout life and potentially reversible (Szyf et al., 2008).

There are several mechanisms of epigenetic regulation; however, in the central nervous system, the most studied are (Graff et al., 2011): (1) DNA methylation, (2) modification of histones, (3) chromatin conformation, and (4) microRNA (miRNA) regulation. Methylation is the incorporation of a methyl group (CH3) in DNA and occurs in the genome of both prokaryotic and eukaryotic organisms (Jaenisch and Bird, 2003). In multicellular eukaryotes, the methylation of cytosine bases prevents the binding of some transcription factors from affecting the state of chromatin proteins by recruiting MBPs (methyl-CpG-binding proteins), causing an inhibition of gene expression and promoting the repressed state of chromatin (Klose and Bird, 2006). DNA methylation plays a role in cell differentiation and provides a mechanism through which the genome can express multiple phenotypes in a multicellular organism; however, it can also serve as a form of biological adaptation to a constantly changing environment, especially during the first years of life (Szyf, 2012). Histones are proteins that package and organize DNA that also participate in the regulation of chromatin compaction. The modifications that histones are subjected to are acetylation, methylation, phosphorylation, and ubiquitination, among others, affecting chromatin condensation and 3D conformation (Sterner and Berger, 2000).

The epigenetic mechanisms through which ncRNAs regulate gene expression involve several RNA types, such as lncRNA and microRNAs (Wei et al., 2017). lncRNAs are molecules over 200 nucleotides in length that regulate the condensation of chromatin, whereas miRNAs are short sequences (21–25 nucleotides) of RNA that modulate RNA silencing and post-transcriptional regulation of gene expression. miRNAs are widely activated in neurons and are associated with neurogenesis and neuroplasticity processes; moreover, they can play a role in the pathogenesis of depression, thus allowing new treatments to be developed (Dwivedi, 2014).

The 3D arrangement of chromatin is controlled by a combination of factors, including several regulatory proteins such as transcription factors or repressors, long non-coding RNAs (lncRNAs), histone modifications, and the action of remodeling complexes.

The position of genes within the nuclear context is very important for gene expression. For instance, large regions of the genome known as Lamina-Associated Domains (LADs) associate with the nuclear lamina at the periphery of the nucleus. LADs represent up to 35% of the nuclear genome identified as silent chromatin (Guelen et al., 2008). Other genome regions known as Nucleolus-Associated Chromatin Domains (NADs) comprise several megabases of silent chromatin associated with the nucleolus (Nemeth et al., 2010; van Koningsbruggen et al., 2010). The 3D arrangement also includes a dynamic looping architecture that facilitates the fine-tuning of gene expression by controlling contacts between *cis*-regulatory elements, sometimes located in distant parts of the genome. Hence, the three-dimensional arrangement of chromatin is very dynamic and undergoes major changes related to cell activities such as gene expression. However, a more detailed treatment of 3D chromatin arrangement is beyond the scope of this review.

Most epigenetic DNA modifications are reprogrammed during gametogenesis and in the pre-implantation embryo. These events ensure genome-wide removal of methylation in the primordial germ cells and the establishment of sex-specific methylation patterns in the sperm and oocyte. After fertilization, most of the epigenome is erased, with the exception of imprinted differentially methylated regions (DMRs), which results in the generation of the epigenetic profile needed to ensure the pluripotency of the embryo. It is believed that trans-acting factors could discriminate imprinted DMRs from other methylated regions in paternal and maternal genomes. Nevertheless, through mechanisms not yet understood, some epigenetic changes different than DMRs can be passed on from one generation to another, for example, through replication of methylation patterns in the synthesis of new histones (Martin and Zhang, 2007). In animal models, it has been observed that chronic and unpredictable maternal separation induced depressive behavior in the offspring during adulthood, changing the profile of DNA methylation, which is transmitted to the next generation with the consequent alteration in gene expression (Franklin et al., 2010). For instance, early maltreatment in rats produces persistent changes in the methylation profile of the BDNF gene and, consequently, in its expression in the PFC, an effect also found in their offspring (Roth et al., 2009). Therefore, the appropriate regulation of these complex mechanisms is necessary for achieving a normal phenotype that guarantees adequate physical and mental health.

Several factors of the social environment, especially those related to parental care during infancy and stress, can cause significant effects on neurobiological development by altering epigenetic programming, generating long-term consequences on mental health (McGowan and Szyf, 2010; Thayer and Kuzawa, 2011; Sasaki et al., 2013). It is known that the quality of parental care may determine the activation of certain genes in progeny associated with the development of specific brain areas such as the hippocampus, which are involved in regulating the stress response (Meaney, 2001).

In animal models, it has been shown that maternal behavior can trigger a cascade of neurobiological signals capable of activating certain transcription factors like growth factor-inducible protein A (NGFI-A), involved in the recruitment of histone acetyltransferase that acts on chromatin, facilitating demethylation and finally causing an epigenetic reprogramming in the offspring (Szyf et al., 2007). At the experimental level, the maternal care model has been widely used in rats, because care behaviors like licking/grooming (LG) and arched-back nursing (ABN) are easily measurable (Lutz and Turecki, 2014). There is evidence of increased methylation (hence more inactive chromatin and therefore lower transcription) in promoter regions of the gene for glucocorticoid receptor (GR) in the hippocampus of adult rats reared by mothers with low levels of LG-ABN. In contrast, offspring of mothers with high LG-ABN show increased hippocampal expression of GR and therefore the possibility of a greater negative feedback in the HPA axis and lower reactivity to stress (Weaver et al., 2004). Interestingly, changes in methylation patterns induced by the type of maternal care in rats can be reversed in adult life. This can be achieved experimentally through a model of cross fostering in which biological offspring of rats with Low LG are raised by foster mothers with High LG, which causes newborns to become indistinguishable from biological offspring of High LG rats in the methylation pattern of exon 17 GR promoter (Weaver et al., 2004). These findings are relevant because they point to the reversibility potential of induced early programming environments on the molecular machinery and their effects on the phenotype.

Another regulatory molecule of the HPA axis is Arginine Vasopressin (AVP). It acts by enhancing the action of the Corticotropin Releasing Hormone (CRH) under sustained stress situations. In rats exposed to early stress (periodic separation of mother and calf), increased secretion of corticosterone is observed accompanied by a persistent increase in AVP neurons of the paraventricular nucleus of the hippocampus. This in turn is associated with hypomethylation in the regulatory region CGI3 (Murgatroyd et al., 2009).

In humans, prenatal exposure to a depressed/anxious maternal mood has been linked to an increase in gene methylation of the GR gene (NR3C1), which in turn has been associated with an increased salivary cortisol response to stress at 3 months after birth (Oberlander et al., 2008). Furthermore, in suicidal patients with a history of sexual abuse, researchers have observed an increase in methylation of exon 1F NR3C1 and a decrease in its hippocampal expression (McGowan et al., 2009). This suggests that the intergenerational transmission of vulnerability to psychopathology in adulthood may be mediated by early epigenetic modifications (due to an adverse environment) related to the regulation of the stress response. In addition, epigenetic modifications have also been reported as a result of acute psychosocial stress. For example, a study (Unternaehrer et al., 2012) demonstrated the presence of dynamic short-term changes in the pattern of DNA methylation of the oxytocin receptor (OXTR) in blood cells after administration of the Trier Social Stress Test (TSST).

In subjects who report various adverse events during their childhood, including physical, emotional, and sexual abuse, a correlation has been found between the number of adverse events reported and methylation of a specific site of exon 17 of the GR gene. Additionally, this pattern is correlated with the presence of borderline symptoms (Radtke et al., 2015).

A low level of maternal care in childhood, measured with the Parental Bonding Instrument (PBI), is associated with increased methylation of the OXTR and BDNF genes in adult life (Unternaehrer et al., 2015).

In a sample of adopted subjects, the presence of unresolved trauma or loss was evaluated through a semi-structured interview, the Adult Attachment Interview (AAI) and the genotype and methylation levels of the 5HTTLPR locus was determined. Higher levels of methylation in the promoter region are associated with increased risk of unresolved trauma in carriers of phenotype I/I (“long alleles,” usually regarded as “protective”). On the other hand, subjects homozygous for the “short alleles” s/s are likely to display a higher level of unresolved trauma if methylation exhibits lower levels. This finding suggests that the effect of the genotype on unresolved trauma is modified by environmentally induced methylation patterns (van Ijzendoorn et al., 2010).

Comparing subjects with MDD who reported low levels of maltreatment and subjects with BPD with high levels of child adversity (sexual abuse and emotional or physical neglect) through the CTQ (Child Trauma Questionnaire), using methylation analysis of whole genome, revealed differences in the methylation patterns of several genes when considering either diagnoses or levels of abuse. An important result from a biological point of view was the higher methylation of the region cg04927004 MicroRNA gene, MiR124-3. As noted before, micro RNA are short regions of RNA that regulate protein synthesis, are widely expressed in neurons, and are associated with neurogenesis and neuroplasticity processes. Additionally, micro RNA could regulate the expression of genes related to the HPA axis such as NR3C1, whose methylation status has been correlated with BPD (Perroud et al., 2011).

A study with a sample of 24 patients with BPD and 11 control subjects, using pyrosequencing of promoter regions of 14 neuropsychiatric genes, found that average methylation was 1.7% higher in BPD subjects. Additionally, an increase in methylation on CpG sites of 5 genes associated with neurotransmission and stress response (HTR2A, MAOA, MAOB, NR3C1, and S-COMT) was found (Dammann et al., 2011).

Recent research has made it possible to explore the status of methylation across the entire genome: the so-called epigenome-wide association studies (EWASs). This method has revealed, for example, an association between depressive symptomatology and the methylation of genes related to the G-protein coupled receptor signaling pathway (Shimada et al., 2018) and an association between maternal stress and 95 CpG sites including poly(ADP-ribose) polymerase I, an enzyme related to stress signaling (Wright et al., 2017).

The activation of epigenetic processes allows social and environmental experiences, both positive and negative, to produce persistent behavioral changes and be associated with the risk of PDs (Slavich and Cole, 2013). However, is it possible to postulate a relationship between these molecular mechanisms and our psychic life? If so, does psychotherapy play a role?

## Subjective Processes Affect Molecular Mechanisms

Psychotherapy is closely connected to the mental world. In our clinical work we are permanently evaluating and interpreting the first-person accounts that our patients bring (Kendler, 2005). Neuropsychiatry has shown that brain changes produce mental change, but the opposite, that is, that changes in the mind produce changes in the brain, is a recent discovery. Kandel (1999, p. 519) asserted that “insofar as [psychotherapy] is successful in bringing about persistent changes in attitudes, habits, and conscious and unconscious behavior, it does so by producing alterations in gene expression that produce structural changes in the brain.” If genes and the environment interact in the brain shaping each individual brain, then the mind (i.e., subjective experience) plays an inescapable role in this interaction. For the purpose of our review, it is important to address the question of whether and how subjective processes, as mediators/moderators of environmental changes, modify the molecular machinery and determine phenotypic adaptations to the environment.

Kendler (2005) proposes that subjective or “first-person” experiences have causal efficacy in the body and can be understood as highly elaborate forms of intentional processes that eventually lead to action and result in achievements such as language, customs, technology, and culture. Mental disorders emerge from the failure of these intentional states to exert effective action in the world (Spence, 1996). In this regard, Fonagy (2003, p. 108) argues that “*Intrapsychic representational processes are not just consequences of environmental and genetic effects – they may be critical moderators*^1^. […] the primary evolutionary function of attachment may be the contribution it makes to the creation in the individual of a mental mechanism that could serve to moderate psychosocial experiences relevant to gene expression.” In other words, he states that the *interpretation* of the social environment and not the mere physical environment acts on genetic expression.

The subjective perception of the social environment (e.g., perception of isolation or social anxiety) can generate changes in several levels of the body’s response systems, such as the central nervous system, hypothalamic pituitary adrenal axis, intracellular signals, and finally transcription factors and genetic expression. This causal trajectory is known as “Social Signal Transduction” (Slavich and Cole, 2013). An example of this is that perceived social rejection in adolescents predicts increases in inflammatory molecules (NF-κB and I-κB). Faced with a threat to their position in the social hierarchy, molecular mechanisms for responding to a potential physical aggression are activated; this initially adaptive response causes a collateral increase in the risk of cardiovascular and affective disorders (Murphy et al., 2013).

Passing on learning from one generation to another constitutes another mechanism for the transmission of information relevant to survival, in parallel with the transmission of genetic material (Fonagy and Allison, 2014). At the same time, epigenetic modifications can be an articulating mechanism between both forms. The “Social Brain Network” (dorsal medial PFC, temporoparietal junction, posterior superior temporal sulcus, and anterior temporal cortex) is associated with socio-cognitive processes such as mentalizing, social emotion, and peer evaluation, and remains in development until early adulthood (Blakemore and Mills, 2014). Epigenetic mechanisms are both permeable to environmental influences and can be stable over time. Moreover, studies on genome-wide DNA methylation variability in adolescent monozygotic twins suggest that “the methylome remains dynamic in adolescence” (Levesque et al., 2014), so it is possible to argue that they can be a mechanism for long-term effects of both early experiences and significant emotional experiences, such as psychotherapy, in sensitive periods of life. All this is highly relevant if we wish to understand how psychotherapy impacts the molecular level and, construct an *evidence-based explanation* of the mechanisms responsible for change, and determine how these mechanisms operate to produce symptom improvement.

## Epigenetic Changes and Psychotherapy: What Is the Evidence?

We know that the origins of mental illness are linked to environment-genome interaction and that this interaction depends on epigenetic mechanisms (Heim and Binder, 2012). Considering that psychotherapy is a type of treatment that involves learning from the environment (determined by the therapeutic relationship), it is possible to argue that these changes depend on epigenetic modifications. It has even been suggested that psychotherapy could be regarded as an “epigenetic drug” (Stahl, 2012). However, just a few studies have addressed the potential link between epigenetic changes and the effect of psychotherapy and so far no systematic reviews have examined the relationship between psychotherapy and epigenetics. Our hypothesis is that, as the environment produces biological changes that result in epigenetic modifications, psychosocial interventions can have a similar effect.

To test the plausibility of our hypothesis, we conducted a review selecting empirical studies published in peer reviewed journals in English until September 2017, using several databases (PubMed, ScienceDirect, and Medline PsycInfo) plus a manual search using the following terms: epigenetic, acetylation of histones, DNA methylation, chromatin modification, psychosocial and psychotherapy. Journals were selected using the following criteria: (1) empirical studies in human studies; (2) studies included at least one epigenetic measure; (3) studies included any psychotherapeutic or psychosocial intervention.

Only five studies met the selection criteria. Perroud et al. (2013) examined 115 outpatients (and 52 controls) diagnosed with BPD exhibiting suicidal behavior or para-suicidal impulses and uncontrolled hostility. All received intensive dialectical behavior therapy (4 weeks) plus drug treatment, which remained unchanged during the period of application of the psychotherapy in most subjects and was controlled for in the statistical analysis. In addition to personality assessment through the Screening Interview for Axis II Disorder (SCID-II), depressive symptomatology was measured using the Beck Depression Inventory-II (BDI-II), the French version of the Diagnostic Interview for Genetic Studies (DIGS) was used to assess Axis I Disorders, the Beck Hopelessness Scale (BHS) was employed to evaluate negativism and pessimism about the future, the Barratt Impulsiveness Scale (BIS-10) was used to analyze impulsivity, and histories of childhood trauma were estimated with the Childhood Trauma Questionnaire (CTQ). DNA extraction was performed on blood leukocytes. Before and after psychotherapeutic intervention, the percentage of CpG methylation of exons I and IV of the gene brain-derived neurothophic factor gene (BDNF) protein were measured. The study showed that, compared to controls, subjects diagnosed with BPD have a significantly higher state of methylation (directly proportional to the number of traumatic events in childhood) in both regions of the BDNF. In addition, a positive association between BDNF methylation status and level of depression, hopelessness, and impulsivity was found. In patients with BPD, BDNF methylation increased significantly after psychotherapeutic intervention, especially in those not responding to treatment. Those who responded to treatment showed a decrease in the percentage of methylation. Changes in methylation status were significantly related to changes in depressive symptoms, hopelessness, and impulsivity. No association between plasma levels of the BDNF protein and methylation status was found. In order to analyze the effect size, considering that in this study were used percentages (proportions), we computed the Cohen’s *h* index. The effect size of the percentage of methylation (mean of BDNF CpG exon I and BDNF CpG exon IV regions IV) for >75% BDI responders is *h* = 0.77 (“Large”) and for >50 to <75% responders is *h* = 0.53 (“Medium”). The same behavior is observed for Hopelessness. Non-responders (<25%) maintain BDI and Hopelessness levels.

A second study (Yehuda et al., 2013) evaluated 16 veterans with posttraumatic stress disorder (PTSD) in order to determine whether methylation of cytosine in the promoter region of GR gene NR3C1 and the FK506 binding protein 5 (FKBP5) gene (that codes for a co-chaperone protein of the GR) predicts response to prolonged exposure to psychotherapy (12 weeks). The methylation level of DNA extracted from blood lymphocytes before treatment at the end of psychotherapy and at 3-month follow-up was measured. The group was divided into responders and non-responders according to the presence or absence of PTSD criteria measured through the Structured Clinical Interview for DSM-IV (SCID), the Clinician Administered PTSD Scale (CAPS), and the PTSD Symptom Scale-Self Report. NR3C1 gene methylation predicted treatment response, but did not change significantly over time. Patients who had a higher methylation before treatment had a better response to intervention. The result of significant discrimination between responders and non-responders at pre-treatment for methylation of the GR gene (NR3C1) exon 1F promoter (we computed Cohen’s *d* = 3.2 for % of methylation and 3.3 for number of methylated sites) has high statistical power. In fact, for Sawilowsky (2009), these values (higher than 2) are categorized as “Huge.” FKBP51 gene methylation did not predict response to treatment, although it tended to decrease in patients who responded to treatment. In this study, the authors propose that psychotherapy is a form of “environmental regulator” that affects epigenetic states.

In a sample of 56 subjects, patients with a panic disorder diagnosis exhibited lower methylation compared to controls in the monoamino oxidase A (MAOA) gene, which codes for an enzyme that catalyzes the oxidative deamination of amines such as dopamine, norepinephrine, and serotonin. After 6 weeks of CBT, an increase in MAOA methylation correlates with agoraphobia symptom reduction (Ziegler et al., 2016). Because they used ANOVA analysis, effect size was estimated using partial eta squared ($\eta_{p}^{2}$). At baseline, they reported significant 11 from 13 CpG islands. Thus, for the most significant the island, CpG13 with *p*-value < 0.001 and $\eta_{p}^{2}$ = 0.369 (Cohen’s *d* = 6.28 “Huge”), while for the significant islands showing the highest *p*-value, CpG4, the *p*-value is 0.049, $\eta_{p}^{2}$ = 0.108 and *d* = 3.32 “Huge.” After therapeutic intervention, when compared with baseline, they reported as significant 8 from 13 CpG islands and it high-lights that CpG3 maintains high significance (*p*-value = 0.001, $\eta_{p}^{2}$ = 0.446, *d* = 2.9 “Huge”), CpG4 increases (*p*-value = 0.003, $\eta_{p}^{2}$ = 0.365, *d* = 3 “Huge”), while CpG13 and CpG12 become non-significant (*d* = 0.19 “Small”).

Another study with 98 children with anxiety disorders who completed 12 weeks of CBT found that patients with the greatest reduction in anxiety – even those who carry the risk genotype – displayed decreased methylation levels of CpG IV of FKBP5 (Roberts et al., 2015). Percentage DNA methylation at the FKBP5 and GR promoter regions was measured before and after CBT. Statistical analysis considered a mixed linear model and reported that change in CpG4 site DNA methylation of FKBP5 was significantly associated with “good” treatment response (β = 0.04, *P* = 0.0069), which do not allow compute the effect size.

In spite of not being a study that exclusively explores psychotherapy as treatment (Kahl et al., 2016), increased methylation of GLUT 1 – a gene that codes for the insulin independent glucose transporter 1, which is involved in brain metabolism – was found in a sample of 52 depressed patients in comparison to 18 healthy subjects. Additionally, depressed subjects whose depressive symptomatology was in remission after treatment (6 weeks of inpatient treatment, CBT, and antidepressants) showed significantly lower GLUT 1 methylation compared to non-remitters. This result hints at the role of brain glucose metabolism dysfunction in the development and maintenance of depression. They used a mixed model with variables methylation; fixed factors: CpG position; depression group; interaction between CpG position and depression group to assess. As result they obtained F statistic and *p*-value for each effect, deducing as significant the difference between depressed and control groups for baseline GLUT1 promoter methylation [*F*(1,540) = 4.72; *P*-value = 0.030]. From these values we computed the effect size by $\eta_{p}^{2}$ = 0.008 (considered “Small”). The comparison between remitters and non-remitters for GLUT1 methylation results in a significant effect of remission [*F*(1,268) = 15.73; *P* < 0.001; then $\eta_{p}^{2}$ = 0.06 “Medium”] and of baseline GLUT1 methylation levels [*F*(1,268) = 15.70; *P* < 0.001; then $\eta_{p}^{2}$ = 0.06 “Medium”]. Remitters decrease GLUT1 methylation to levels similar to controls.

With few exceptions, effect sizes are high, so significance of discoveries about association between treatment-responses and epigenetic results strengthened.

The exploration of the epigenetic mechanisms that may underlie psychotherapeutic changes is a budding area of research. Multiple aspects must be refined and limitations must be overcome, such as increasing sample sizes, homogenizing both phenotype and type of psychotherapy, including healthy controls to assess whether variations in methylation are due to the mere passage of time, and controlling for confounding environmental factors such as the use of tobacco and psychiatric drugs. Another aspect to consider is the duration of the psychotherapies; in general, in the studies presented they have not exceeded 12 weeks, which may be insufficient to cause persistent changes, for instance, in personality functioning (Lindfors et al., 2015). Finally, studies in this area must begin using peripheral tissues as a proxy to evaluate molecular changes at the brain level.

It is not possible to use *in vivo* brain tissue for epigenetic research. On the other hand, post-mortem studies using brain tissue are useful, but also have limitations such as temporal discordance between phenotype development and time of analysis, changes in methylation patterns due to causes of death, and small sample sizes (Bakulski et al., 2016). This makes peripheral blood and buccal cells the preferred tissue types for psychiatry research, given the ease of non-invasive specimen collection and the possibility of obtaining larger samples. Despite these advantages, their use involves a number of considerations and limitations (for exhaustive reviews of the subject see Bakulski et al., 2016). Principal component analysis revealed that the most important component in explaining variance in methylation levels is precisely tissue type (Farre et al., 2015). Another aspect to consider is the existence of a different type of DNA modification called hydroxymethylation, a very active process in the brain, but rare in the blood. The usual methods for determining methylation levels fail to distinguish between the two (Wen et al., 2014). The use of whole blood has the disadvantage of cellular heterogeneity. Algorithms for estimating cell types and creating blood cell reference panels for different populations can help overcome these limitations (Bakulski et al., 2016).

In support of the use of tissues other than the brain, there is preliminary evidence of correlation between brain tissue and peripheral blood. For example, in a study of samples of temporal lobe biopsies from patients with epilepsy and peripheral blood from healthy subjects and schizophrenic patients, the DNA methylation analysis showed a 7.9% correlation between blood and brain, a relatively low percentage, but significantly higher than expected by chance (Walton et al., 2016). Other studies show evidence of a correlation between the BDNF methylation patterns of muscle tissue and those of PFC tissue in a postmortem study in humans (Stenz et al., 2015); also, experimentally, concordance was found between brain and blood of mice and human cord blood (Kundakovic et al., 2015). In relation to FKBP5, mice exposed to corticosteroids exhibit changes in methylation in both hippocampus and blood (Ewald et al., 2014). These findings, although very limited, suggest that the DNA methylation patterns of blood cells could be used as biomarkers of stress-induced central nervous system responses.

## Discussion: Toward a Psychotherapeutic Treatment Based on the Mechanism of Disease

Since Freud (2001) set out to construct a “natural science of psychology” based on the study of quantifiable psychic processes, the surprising advance of scientific knowledge has made it possible to reveal not only neurobiological mechanisms underlying mental functioning, but also the intricate relationships that exist between genes and the environment, where epigenetic regulation processes play a fundamental role.

Although the problem of multiple causality in psychiatry is not new (Jiménez, 1979) it is now evident that understanding complex psychological phenomena such as mental illness requires a perspective that includes multiple levels of analysis (Kendler, 2012), from genes to behavior, including brain structures, the functioning of specific areas such as the amygdala, cognitive processing, and emotional states such as distress or depression. We suggest that the same kind of multilevel analysis proposed for psychiatry should be applied to the study of change in psychotherapy. Multilevel analysis moves away from biological or psychological reductionism to embrace epistemological pluralism. For decades, explanatory theories of psychopathology have overlooked the fact that human beings are mind-brain units. The construction of a scientific psychology proposed by Freud has encountered the ‘difficult problem’ that the world of meaning cannot be reduced to molecular mechanisms. Nevertheless, a pluralistic approach will allow us to further our understanding of the biological mechanisms involved in psychotherapeutic change, beyond the psychological sphere.

We know that the origins of mental illness are linked to the environment-genome interaction and that this interaction depends on epigenetic mechanisms (Heim and Binder, 2012). On the other hand, we also know that psychotherapy is effective (Lambert, 2013b), that its results depend largely on non-specific factors (Wampold and Imel, 2015) related to interpersonal processes (Mitchell, 1988; Stolorow, 2004), and that it produces biological changes in the central nervous system (Barsaglini et al., 2014).

Both the early interaction with caregivers and interpersonal experiences later in life allow individuals to acquire new strategies for processing the social environment, in accordance with the demands of the context and the individual’s stage of development, favoring adaptation. Epigenetic changes emerge as a possible mechanism for transforming this new information into a more or less stable reconfiguration of neural systems and finally achieving a better phenotypic adaptation. It is precisely at the interface between the environment and our genes where epigenetic processes occur and can be understood as a way to biologically encode the impact that life events have on an organism (Isles and Wilkinson, 2008), serving as a molecular bridge between “nature” and “nurture” (Tammen et al., 2013). In that regard, we may think that epigenetic mechanisms are the biological way in which the environment is internalized and becomes part of what psychoanalysts call the subject’s internal reality. It can be argued that this is possible since the processes of activation and suppression of gene activity, such as methylation, have the property of being sensitive to environmental stimuli while remaining stable over time. In this regard, Levesque et al. (2014), studying epigenetic changes in the complete genome in adolescents, propose that two groups of genes exist: “state genes,” whose patterns of methylation are highly variable, being capable of changing in months, and “trait genes,” which are permeable to environmental influences but stable over time.

Kandel (1998, 1999) conceptualized psychotherapy as a type of learning dependent on environmental influences, associating its neurobiological effect with the expression of certain genes related to the functioning and structure of synaptic connections in the brain. If this is so, from a biological point of view, psychotherapeutic changes depend on epigenetic modifications. However, although a significant amount of evidence exists regarding the biological effects of psychotherapy, only a few studies have examined the epigenetic mechanisms underlying this effect. On the other hand, it is also striking that, despite the abundant information about the impact of the psychosocial environment on the genome, so few studies have analyzed the effect of psychotherapy on the genome.

In our review, we found only five studies about the relationship between epigenetics and psychotherapy. The studies reported looked at only five genes (BDNF, NR3C1, FKBP51, MAOA, and GLUT1) related to the stress system, neurotransmission, neuroplasticity and brain metabolism, and PTSD, BPD, panic disorder, MDD, and anxiety in children as phenotypes. In addition, brief psychotherapeutic interventions made it difficult to evaluate the stability of epigenetic changes over time. The studies identified have different objectives in terms of the relationship between psychotherapy and epigenetics: while some assessed epigenetic modifications that occur after psychotherapy, others studied epigenetic changes before treatment implementation.

With the information available so far, it is hard to assess the potential clinical impact of the study of epigenetics and psychotherapy; however, the analysis of epigenetic changes can help identify biomarkers for improving diagnosis, also opening up future research possibilities regarding the mechanism of action of antidepressant drugs (Dalton et al., 2014) and psychotherapy. Early traumatic experiences produce epigenetic modifications in neurodevelopmental genes that are related to adult psychopathology (especially BPD) and can be modified by psychotherapy. Taking into account the limited evidence available, some PDs such as BPD and panic disorder exhibit distinctive patterns of gene methylation associated with functions of neurotransmission or neuroplasticity. Preliminary evidence indicates that these methylation profiles may moderate the effect of psychotherapy or change as a function of the patient’s response to it. Even the study of certain epigenetic changes (such as methylation level) could be used as a predictor and indicator of response to psychotherapy. Although so far it has only been hypothesized, the pharmacological enhancement of learning and memory through epigenetic modifications could boost the effect of psychotherapy and long-term rehabilitation in diseases of the central nervous system (Gavin et al., 2011).

Children inherit not only genes from their parents, but also a coded environment in them. Given that there is some evidence for the transgenerational transmission of epigenetic modifications in humans exposed to traumatic situations (Yehuda et al., 2016), it is possible to hypothesize that epigenetic changes produced by psychotherapy could also potentially be passed on to offspring. Additionally, the fact that epigenetic changes are reversible may be an argument for reinforcing the indication of psychotherapy.

In addition to the dynamic changes of the genome, the recognition of other sources of variability such as genetic polymorphisms could make it possible to identify subjects who, according to the model of differentiated sensitivity, are particularly receptive to positive environmental stimuli and may respond better to psychotherapeutic interventions. In fact, the available evidence supports the notion that the effect of interventions is greater in genotypes considered to be susceptible than in non-susceptible ones (Bakermans-Kranenburg and van van IJzendoorn, 2015). Consequently, the analysis of these biological variables could be useful as an indicator of response and, therefore, prognosis for psychotherapy.

Jablonka and Lamb (2005) use the concept of “socially mediated learning,” that is, learning how adults behave to ensure survival and mating. In the case of human beings, in addition to behaviors, it is possible to transmit information symbolically through language, which constitutes a new system of non-genetic inheritance. The same authors argue that the different dimensions of inheritance – genetic, epigenetic, behavioral, and symbolic – interact with one another in the configuration of the phenotype. The transmission of information about how to navigate the social world from those perceived to be reliable (caregivers and peers) enables individuals to efficiently reap the benefits of community life, i.e., the construction of so-called “epistemic trust” (Fonagy and Allison, 2014). Psychotherapy can act by “recalibrating” systems of sensitivity to the social environment, for example by increasing the reward value of interpersonal relationships, improving the quality of bonding, and indirectly reducing anxiety and depressive symptoms regardless of the specific disorder (Quevedo, 2016).

One of the major challenges posed by research into complex phenomena such as PDs or change in psychotherapy is how to incorporate the inherent complexity of these phenomena into their methods and the interpretation of their findings without losing their heuristic value (Cacioppo and Decety, 2011). Moreover, given the growing evidence of how external social conditions and especially our subjective experience of them can influence a number of internal biological processes (Slavich and Cole, 2013), the study of the intimate relationship between genes and the environmental context is of particular relevance. Although it has been suggested that psychotherapy could be regarded as an “epigenetic drug” (Stahl, 2012), there is still a long way to go before we manage to understand the biological mechanisms on which interventions are based. For psychotherapy research, this field can be particularly fertile especially if we consider that change results not only from specific psychotherapeutic techniques in the session, but also from the capacity of the therapeutic relationship to promote learning about oneself and others outside the framework of the session, that is, in the social environment where the individual develops (Fonagy et al., 2015). This will be achieved if we conduct studies that integrate the complex relationships between levels of analysis, including variables such as personality, subjective experience, and culture.

## Recommendations for Future Research

We should be cautious when considering the state of current research in epigenetics and psychopathology, in particular methylation studies, since this is a developing field that must contend with a number of limitations. Moffitt and Beckley (2015) list some of them, namely (1) The small environmental effect size expected on the methylation pattern, which is predominantly determined by the programming of cellular differentiation, (2) The specificity of the methylation patterns of each tissue and cell population, (3) Our current ignorance of the most dynamic regions and those with the most sensitivity to the social environment, (4) The need for new statistical approaches and laboratory techniques for processing whole epigenome data, (5) The need to clarify the link between methylation and actual changes in gene and phenotypic expression, and finally (6) The risk of falling into deterministic thinking when interpreting results.

A summary of recommendations for future research is shown in **Table 1**.

**Table 1:** Recommendations for future research.

(1) It is necessary to consider that epigenetic modifications are influenced by multiple environmental variables (such as exercise, diet, or drug use) that may interfere with the assessment of changes produced by psychotherapy.
(2) Because epigenetic changes can vary over a lifetime and even be reverted, long-term studies incorporating a life-cycle approach would be useful.
(3) In addition, it would be interesting to determine the specificity of epigenetic change in psychotherapy. For that purpose, it will be necessary to define and justify with precision both the expected epigenetic changes and the environmental factors that will be studied, describing the psychobiological model in which they are included.
(4) It may be useful to study intermediate phenotypes or endophenotypes such as certain cognitive attributes, personality traits, or the functioning of differentiated neurobiological systems.
(5) It is also necessary to conduct studies with adequate explanatory power, with advance registration of target genes and analysis strategies, and with a focus on transdiagnostic domains of functioning.
(6) Research should incorporate models of “plasticity” and “differentiated susceptibility” in order to measure not only the presence/absence of disease or vulnerability to the environment, but also the potential moderating influence of positive factors such as social support or subjective well-being.
(7) Finally, we highlight the need for multilevel studies that include complex relationships between variables (gene–gene, gene–environment, gene–culture).


## Author Contributions

JJ and AB developed, organized, and wrote the manuscript and formulated the conclusions. CL contributed wrote the nature–nurture relationship section. LH and FM contributed to the writing of the gene expression changes section. YQ first drafted the subjective processes and epigenetics section. JJ, AB, JR, JS, LS, UR, RA, and MO designed and outlined the initial idea of the article. PB, KJ, and PL contributed to the final editing of the text. JJ and PL obtained funding (described below). All authors contributed to manuscript revision. All authors read and approved the submitted version.

## Conflict of Interest Statement

The authors declare that the research was conducted in the absence of any commercial or financial relationships that could be construed as a potential conflict of interest.

## Footnotes

1 Italics in original.
